# Temporal dynamics in the parasite communities of corocoro grunt *Orthopristis rubra* (Actinopterygii: Haemulidae) from Sepetiba Bay, southeastern Brazil: a comparative study from 1991 to 2024

**DOI:** 10.1590/S1984-29612026012

**Published:** 2026-05-25

**Authors:** Adriana Rodrigues Mainenti, Fabiano Paschoal, Thiago dos Santos Cardoso, Raquel de Oliveira Simões, José Luis Luque

**Affiliations:** 1 Universidade Federal Rural do Rio de Janeiro – UFRRJ, Programa de Pós-graduação em Biologia Animal, Seropédica, RJ, Brasil; 2 Universidade do Estado do Rio de Janeiro – UERJ, Departamento de Ciências Médicas Integradas, Cabo Frio, RJ, Brasil; 3 Instituto Oswaldo Cruz - Fundação Oswaldo Cruz – FIOCRUZ, Laboratório de Biologia e Parasitologia de Mamíferos Silvestres Reservatórios, Rio de Janeiro, RJ, Brasil; 4 Universidade Federal Rural do Rio de Janeiro – UFRRJ, Departamento de Parasitologia Animal, Seropédica, Rio de Janeiro, Brasil

**Keywords:** Ecological indicators, environmental recovery, Orthopristis rubra, parasite communities, Sepetiba Bay, temporal variation, Indicadores ecológicos, recuperação ambiental, Orthopristis rubra, comunidades parasitárias, Baía de Sepetiba, variação temporal

## Abstract

This study investigated the temporal dynamics of metazoan parasite communities of the corocoro grunt *Orthopristis rubra* (Haemulidae) from State of Rio de Janeiro, Sepetiba Bay, southeastern Brazil, comparing samples collected in 1991–1992, 2011–2012, and 2022–2024. A total of 180 fish were examined, revealing 26 parasite taxa belonging to seven higher groups. The helminth community showed aggregated distributions and a clear temporal pattern in abundance and diversity. Multivariate analyses (nMDS and PERMANOVA) indicated significant but moderate differences among periods, with increased species richness, evenness, and diversity in recent years. Hill diversity profiles revealed higher Shannon and Simpson indices in 2022–2024, suggesting greater balance and reduced dominance. These trends indicate gradual reorganization of parasite assemblages, likely associated with ecological recovery of Sepetiba Bay. The persistence of core digeneans and the appearance of new taxa reflect shifts in food-web structure and host–parasite interactions. The results reinforce the value of long-term parasitological data as bioindicators of ecosystem change in coastal environments under anthropogenic pressure.

## Resumo

Este estudo investigou a dinâmica temporal das comunidades de metazoários parasitos da corcoroca *Orthopristis rubra* (Haemulidae) na Baía de Sepetiba, Estado do Rio de Janeiro, sudeste do Brasil, com base em coletas realizadas em 1991–1992, 2011–2012 e 2022–2024. Foram examinados 180 peixes, registrando-se 26 táxons parasitários distribuídos em sete grupos. As comunidades parasitárias apresentaram padrão de distribuição agregada e variações significativas na abundância e na diversidade ao longo do tempo. As análises multivariadas (nMDS e PERMANOVA) indicaram diferenças moderadas entre os períodos, com aumento da riqueza, da equitabilidade e dos índices de diversidade nos anos mais recentes. Os índices de diversidade de Hill evidenciaram maiores valores de Shannon e Simpson em 2022–2024, sugerindo comunidades mais homogêneas e com menor dominância. Esses resultados apontam reorganização gradual dos conjuntos de parasitos, possivelmente associada à recuperação ecológica da Baía de Sepetiba. A persistência de espécies centrais e o surgimento de novos táxons refletem alterações na estrutura trófica e nas interações hospedeiro-parasito. Os achados reforçam a importância de séries parasitológicas de longo prazo como bioindicadores das mudanças em ecossistemas costeiros sob pressão antrópica.

## Introduction

The family Haemulidae, popularly known as grunts, represents one of the most diverse groups of fish encompassing about 138 species distributed across 23 genera, with broad global distribution, mainly inhabiting tropical and subtropical marine waters, while occasionally occurring in brackish water but rarely in freshwater (Nelson et al., 2016; Froese & Pauly, 2025). In Brazil, haemulids represents an essential ichthyofaunistic component in marine and coastal ecosystems, playing an important ecological role transporting organic matter from reefs to adjacent areas, and form a key link in the coastal food web (Santos et al., 2007; Nelson et al., 2016; Froese & Pauly, 2025). Commonly known as Corocoro grunt, *Orthopristis rubra* (Cuvier, 1830) occurs from Honduras to Brazil in western coast of the Atlantic (Froese & Pauly, 2025). They feed on invertebrates as crustaceans and mollusks, among others (Cervigón, 1991). The Corocoro grunt is a commercial fish, appreciated for its taste and can be found fresh or cured with salt, and off Rio de Janeiro it stands out as the most abundant haemulid representative, gaining recognition for its significant contributions to community composition studies and its utility as a bioindicator species (Cervigón et al., 1992; Santos et al., 2007; Seixas et al., 2021).

Sepetiba Bay is considered one of the most important aquatic ecosystems in the state of Rio de Janeiro, due to its role as a breeding ground for fish and crustaceans of economic importance (Costa, 1998). The bay is bordered to the north by the Serra do Mar Mountain range, to the northeast by the Baixada Fluminense region, to the southeast by the Pedra Branca massif, and to the south by the Marambaia restinga (Costa, 1998). Since 1950, this bay has been impacted by anthropogenic actions such as urbanization and industrialization. Nowadays, Sepetiba bay harbors ports, industrial park, and has been observed an intensive population growth nearby (Pfeiffer et al., 1985; Araújo et al., 2002).

Biotic and abiotic factors shape the composition and diversity of parasite communities within fish hosts. Host trophic level, size and density can influence parasite species richness and transmission success (Poulin, 2004; Mouritsen & Poulin, 2005) as well as water temperature, salinity, pollutant load and habitat degradation can play critical roles by altering host susceptibility or the survival of free-living parasite stages (Marcogliese, 2008). Therefore, long-term comparative studies tracking parasite communities in fish across decades are essential to understanding the dynamic processes of parasitism over time, including patterns of community assembly, species turnover, and resilience in the face of environmental change (Espínola-Novelo et al., 2020; Virgilio et al., 2023).

Over the last decades, ecological studies conducted in Sepetiba Bay have focused on fish and their parasites (Luque et al., 1996a; Araújo et al., 1998; Araújo et al., 2002; Benicio et al., 2022). Long-term comparative studies of parasite communities in fish are essential to understanding temporal shifts in host–parasite dynamics, as they reveal subtle yet significant patterns of species turnover and community restructuring that static diversity measures may overlook (Soares et al., 2014; Negreiros et al., 2019). The main purpose of this study is to compare the parasite community structure from *O. rubra* collected in Sepetiba Bay in 1991/1992, 2011/2012 and 2022/2024, evaluating alterations in ecological parameters during the last thirty years.

## Material and Methods

### Host collection, processing and analysis

Host samples of this study were obtained directly from licensed fishermen in Sepetiba Bay (22°57′18″S, 43°54′44″W), off the State of Rio de Janeiro, southeastern Brazil. Fish were purchased at a local market and analyzed mostly fresh, but some were kept frozen at -20 °C until examination. A total of 180 specimens of *O*. *rubra* were examined, 60 collected between April 1991 and December 1992 (data previously published by Luque et al., 1996a, b), 60 collected between December 2011 and July 2012 (data previously published by Paschoal et al., 2023), and 60 collected between October 2022 and June 2024. The specimens were identified according to Menezes & Figueiredo (1980); the nomenclature and classification were updated according to FishBase (Froese & Pauly, 2025). Each fish was measured for total length. The difference in body length between the fish samples were compared using analysis of variance (ANOVA), followed by the post hoc test of Tukey, since parasite burdens can be correlated with host length (Zar, 2010).

### Parasitological procedures

Nematodes, trematodes, cestodes and acanthocephalans were collected from the stomach, body cavity, and intestine washed in saline solution (NaCl 0.85%), fixed in AFA (acetic acid 2%, formaldehyde 3% and ethanol 95%) and preserved in 70% ethanol. The nematodes were cleared in lactophenol, the cestodes, trematodes and acanthocephalans were stained with chlorhydric carmine (Eiras et al., 2006). The monogeneans were collected from the gills and pharynx washed in saline solution (NaCl 0.85%), fixed in hot 4% formalin, stained with Gomori’s trichrome and mounted in Canada balsam. Hirudineans and Crustaceans were collected using the procedures described by Madinabeitia & Nagasawa (2012), preserved in 70% ethanol, cleared in 85% lactic acid for morphological observation, and examined using the wooden slide procedure of Humes & Gooding (1964). General identification of helminth parasites was according to Khalil et al. (1994), Bray et al. (2008), Gibbons (2009) and Amin (2013). The identification of crustacean parasites was according to Cressey (1991), Luque & Takemoto (1996) and Boxshall & Halsey (2004).

### Data analysis

To analyze the parasite community structure, prevalence, mean intensity and mean abundance were used as parasite population descriptors (at component population level), according to Bush et al. (1997). The index of dispersion (ID; variance/mean ratio of parasite abundance; where values close to 1 indicate a random distribution, values less than 1 suggest a uniform distribution, and values greater than 1 indicate an aggregated distribution) and the discrepancy index (D; which ranges from 0 = fully random or uniform to 1 = fully aggregated) (Poulin, 1993) were used to evaluate the distribution of parasite species within the host population. The frequency of dominance (percentage of infracommunities in which a parasite species was numerically dominant) was calculated according to Rohde et al. (1995).

The analysis of the helminth community was conducted considering both species abundance and incidence across different collection years. Initially, distances among samples were calculated using the Gower index, chosen because it handles mixed abundance and categorical data (Oksanen et al., 2025). Based on this distance matrix, a Non-metric Multidimensional Scaling (nMDS) analysis with two dimensions was performed to graphically represent the similarity among helminth communities across different collection years. To statistically test differences in community composition among collection years, a PERMANOVA was applied. For each temporal group, convex hull polygons were generated to facilitate the visualization of differences between years.

To explore community diversity, helminth abundances were transformed into presence/absence data, allowing the construction of incidence-based rarefaction and extrapolation curves using the iNEXT package. Diversity curves were calculated for species richness (q = 0), Shannon diversity (q = 1), and Simpson diversity (q = 2), considering the number of hosts sampled as the basis for extrapolation.

In addition to these indices, other diversity metrics were calculated based on abundances, including the Berger-Parker index, the Brillouin index, and evenness. All ecological diversity variables (richness, total abundance, Berger-Parker, Brillouin, and evenness) were subsequently log-transformed to meet normality assumptions. To assess differences in diversity among years, ANOVA was performed for each diversity variable, followed by the Tukey HSD test for multiple comparisons. Linear model results were also summarized in forest plots, highlighting estimated coefficients, confidence intervals, and the statistical significance of differences among years. This approach allowed the evaluation of temporal changes in helminth community composition and diversity, considering both quantitative aspects (abundance and diversity) and qualitative aspects (species presence/absence), as well as specific indices reflecting dominance and evenness.

#### Softwares and packages

All analyses were conducted in R software version 4.5.1 (R Core Team, 2025). The Gower distances, nMDS, PERMANOVA, ANOVA and Tukey test were performed using vegan package (Oksanen et al., 2025), the incidence-based rarefaction and extrapolation curves and calculation of Hill diversity indices were performed using iNEXT package (Chao et al., 2014), plots, including nMDS and forest plot, were performed using ggplot2 package (Wickham, 2016). The level of significance adopted was 5% in all analyses.

## Results

### Characteristics of host samplings

Fish mean total body length was 24.8 ± 5.4 (13.1-35.5) cm. A comparison of total body length revealed that fish samples were statistically significant (F = 104.7, p < 0.001). The samples from 1991–1992 were smaller (19.2 ± 2.5) than those from 2011–2012 (28.1 ± 3.7) and 2022–2024 (27.1 ± 4.5) (Q = 18.7, p < 0.01; Q = 16.4, p < 0.01, respectively). The samples of 2011–2012 and 2022–2024 did not differentiate statistically (Q = 2.3, p > 0.09).

### Characteristics of parasite communities

The total parasite community was composed by 26 different taxa, 15 of which endoparasites belonging to Acanthocephala, Cestoda, Digenea and Nematoda, including larval and adult stages, and 21 ectoparasites belonging to Copepoda, Hirudinea and Monogenea, all adult forms (Table 1). A total of 4052 parasite individuals were collected, with a mean abundance of 22.5 ± 29.2. One hundred and seventy-five specimens of *O*. *rubra* were parasitized by at least one taxon of metazoan (59 in 1991–1992; 56 in 2011–2012; 60 in 2022-2024), and five were negative for parasites (one in 1991–1992; four in 2011–2012). Adult endoparasites were the most abundant group in all samples, corresponding to 69.47% (73.6% in 1991–1992; 65.5% in 2011–2012; 70.3% in 2022–2024) of all parasite specimens collected; followed by ectoparasites with 24.31% (18.4% in 1991–1992; 29.7% in 2011–2012; 23.2% in 2022–2024) and larval endoparasites with 6.21% (7.8% in 1991–1992; 4.7% in 2011–2012; 6.4% in 2022–2024). All parasite species had an aggregated distribution pattern (ID > 1), and overall, they showed discrepancy index (D) values higher than 0.56 (Table 2). When comparing the mean abundance, mean species richness, mean diversity indexes (Brillouin and evenness) and Berger-Parker dominance among samples, significant differences were observed (Table 3). In all communities studied, the digenean *Infundiburictus longovatus* (Hopkins, 1941) was the species with the highest frequency of dominance (Table 3).

**Table 1:** Metazoan parasites of *Orthopristis rubra* according to collection samples, associated with their prevalence (P), mean intensity ± standard deviation (MI±SD), mean abundance ± standard deviation (MA±SD), and site of infection or infestation (Site), from the coastal zone of Rio de Janeiro, Brazil.

**Parasites**	**Sampling of 1991-1992**			**Sampling of 2011-2012**			**Sampling of 2022-2024**			**Site**
	**P(%)**	**MI±SD**	**MA±SD**	**P(%)**	**MI±SD**	**MA±SD**	**P(%)**	**MI±SD**	**MA±SD**	
**Digenea**										
*Aponurus pyriformis*	20	1.9±2.01	0.3±1.1	18.3	2.1±1.2	0.4±0.9	25	3.4±2.2	0.8±1.8	Stomach
*Diphterostomum brusinae*	28.3	3.7±3.05	1.1±2.3	16.6	6.1±11.1	1.01±4.9	21.6	3.7±4.3	0.8±2.5	Intestine
*Diplangus paxillus*	30	38.2±53.7	11.4±33.8	--	--	--	30	2.9±2.2	0.8±.8	Stomach
*Diplomonorchis leiostomi*	--	--	--	11.6	10.5±9.5	1.2±4.5	--	--	--	Intestine
*Genolopa ampullacea*	--	--	--	11.6	4.4±3.2	0.5±1.7	--	--	--	Intestine
*Infundiburictus longovatus*	33.3	10.05±10.4	3.3±7.5	50	26.6±31.7	13.3±26.02	46.6	6.1±8.3	2.8±6.4	Stomach
*Opecoeloides* sp.	3.3	1	0.03±0.1	10	1.8±1.1	0.1±0.6	10	1.5±0.8	0.1±0.5	Intestine
*Postmonorchis orthopristis*	21.6	3.2±2.7	0.7±1.8	--	--	--	20	2.1±1.2	1.1±3.5	Stomach
*Prosorhynchus ozakii*	16.6	3.6±3.9	0.6±2.1	11.6	2.1±1.4	0.2±0.8	33.3	5.9±6.2	1.5±2.9	Intestine
*Torticaecum* sp. (immature)	--	--	--	20	3.4±1.7	0.6±1.5	6.6	1.5±0.5	0.1±0.3	Body cavity
**Monogenea**										
*Choricotyle aspinochorda*	1.66	2	0.03±0.2	--	--	--	11.6	1.7±0.7	0.2±0.6	Gills
*Choricotyle brasiliensis*	6.66	1	0.06±0.2	26.6	1.5±0.8	0.4±0.7	13.3	2.6±1.8	0.3±1.1	Gills
*Choricotyle orthopristis*	1.66	1	0.01±0.1	15	2.1±1.6	0.3±0.9	8.3	2.2±1.6	0.1±0.7	Gills
*Encotyllabe parvum*	60	2.9±1.9	1.6±2.1	78.3	4.9±5.2	3.8±5.03	30	2.7±2.5	0.8±1.8	Pharynx
*Neoheterobothrium cynoscioni*	3.3	1.5±0.7	0.05±0.2	3.3	1	0.03±0.1	--	--	--	Gills
*Pseudotagia rubri*	46.6	4.8±4.7	2.2±4.01	66.6	3.9±2.8	2.6±2.9	18.3	1.9±1.5	0.3±0.9	Gills
**Cestoda**										
Larvae of *Scolex*	15	12.4±17.2	1.8±7.7	--	--	--	6.6	1.5±0.5	0.1±0.3	Intestine
**Acanthocephala**										
*Dollfusentis chandleri*	20	2.3±2.3	0.4±1.3	18.3	1.4±0.6	0.2±0.6	16.6	3.4±2.2	0.5±1.5	Intestine
*Serrasentis* sp.	5	1.3±0.5	0.06±0.3	26.6	2.6±1.5	0.7±1.4	13.3	5.2±2.8	0.7±2.1	Intestine
**Nematoda**										
*Cucullanus* sp.	--	--	--	16.6	1.6±1.2	0.2±0.7	10	2.1±1.9	0.2±0.8	Intestine
*Dichelyne* (*C*.) *tornquisti*	--	--	--	35	4.8±5.8	1.7±4.5	28.3	2.5±2.4	0.7±1.6	Intestine
**Hirudinea**										
Piscicolidae gen. et sp.	5	1	0.05±0.2	--	--	--	3.3	1	0.03±0.1	Gills chambers
**Copepoda**										
*Caligus haemulonis*	15	1.3±0.7	0.2±0.5	41.6	3.1±2.3	1.2±2.1	11.6	3.8±2.6	0.4±1.5	Gills chambers
*Caligus sepetibensis*	6.6	1	0.06±0.2	3.3	1	0.03±0.1	11.6	2.5±1.7	0.3±0.9	Gills chambers
*Lernanthropus rathbuni*	8.3	1	0.08±0.2	10	1.1±0.4	0.1±0.3	20	2.5±2.1	0.5±1.3	Gills
*Parashiinoa* sp.	--	--	--	3.3	1	0.03±0.1	--	--	--	Nostril

**Table 2:** Values of variance-to-mean abundance ratio (ID) and index of Discrepancy (D) of metazoan parasites of *Orthopristis rubra* according to samples, from the coastal zone of Rio de Janeiro, Brazil.

**Parasites**	**Sampling I**		**Sampling II**		**Sampling III**	
	**ID**	**D**	**ID**	**D**	**ID**	**D**
**Digenea**						
*Aponurus pyriformis*	3.55	0.863	2.47	0.855	4.02	0.824
*Diphterostomum brusinae*	5.11	0.809	23.62	0.927	7.69	0.879
*Diplangus paxillus*	99.72	0.879	--	--	3.69	0.801
*Diplomonorchis leiostomi*	--	--	16.97	0.921	--	--
*Genolopa ampullacea*	--	--	6.01	0.907	--	--
*Infundiburictus longovatus*	17.24	0.818	50.86	0.772	14.6	0.749
*Opecoeloides* sp.	--	--	2.31	0.914	1.77	0.909
*Postmonorchis orthopristis*	4.71	0.858	--	--	10.87	0.886
*Prosorhynchus ozakii*	7.07	0.897	2.81	0.906	5.98	0.782
*Torticaecum* sp. (immature)	--	--	3.65	0.839	--	--
**Monogenea**						
*Choricotyle aspinochorda*	--	--	--	--	1.83	0.893
*Choricotyle brasiliensis*	--	--	1.54	0.786	3.47	0.899
*Choricotyle orthopristis*	--	--	3.05	0.889	--	--
*Encotyllabe parvum*	2.63	0.619	6.59	0.594	4.29	0.817
*Pseudotagia rubri*	7.11	0.747	3.38	0.566	2.69	0.864
**Cestoda**						
Larvae of *Scolex*	32.31	0.922	--	--	--	--
**Acanthocephala**						
*Dollfusentis chandleri*	4.03	0.868	1.51	0.842	4.21	0.875
*Serrasentis* sp.	--	--	2.82	0.804	5.97	0.889
**Nematoda**						
*Cucullanus* sp.	--	--	2.27	0.869	3.46	0.922
*Dichelyne* (*C*.) *tornquisti*	--	--	10.08	0.828	4.03	0.806
**Copepoda**						
*Caligus haemulonis*	1.49	0.866	3.49	0.729	5.08	0.909
*Caligus sepetibensis*	--	--	--	--	3.31	0.905
*Lernanthropus rathbuni*	--	--	1.19	0.897	3.71	0.859

Sampling I = 1991-1992; Sampling II = 2011-2012; Sampling III = 2022-2024.

**Table 3:** Descriptors of parasite communities of *Orthopristis rubra* among samples from the coastal zone of Rio de Janeiro, Brazil, represented as mean followed ± 1 standard deviation (range), with the values of ANOVA (F) and the evaluation by *a posteriori* Tukey test (Q).

**Characteristics/ Year**	**S_I_**	**S_II_**	**S_III_**	***F***	***Value of post-hoc tukey* (*Q*)**		
Parasite species richness	20	21	22	--	**S_I_ -S_II_**	**S_I_-S_III_**	**S_II_-S_III_**
Total number of specimens	1472	1753	827	--	--	--	--
Mean species richness ± SD	3.45 ± 1.46 (0-7)	4.91 ± 2.52 (0-11)	3.96 ± 2.20 (1-10)	7.204^*^	5.295*	1.887	3.408^*^
Mean total abundance ± SD	24.53 ± 36.38 (0-188)	29.21 ± 30.07 (0-166)	13.78 ± 14.72 (1-80)	4.581^*^	1.266	2.907	4.174^*^
Mean Brillouin index ± SD	0.62 ± 0.32 (0-1.31)	0.88 ± 0.44 (0-1.60)	0.80 ± 0.46 (0-1.72)	5.852^*^	4.712^*^	3.304	1.408
Mean evenness index ± SD	0.67 ± 0.31 (0-1.01)	0.84 ± 0.28 (0-1.00)	0.79 ± 0.29 (0-1.00)	4.976^*^	4.342^*^	3.058	1.284
Dominant species	*I*. *longovatus* 23%	*I*. *longovatus* 31%	*I*. *longovatus* 20%	--	--	--	--
Mean Berger-Parker Index ± SD	0.62 ± 0.21 (0-1.00)	0.50 ± 0.24 (0-1.00)	0.51 ± 0.21 (0-1.00)	5.576^*^	4.403^*^	3.680^*^	0.723

S_I_ = Sampling of 1991-1992; S_II_ = Sampling of 2011-2012; S_III_ = Sampling of 2022-2024.

* Significant value (P < 0.05).

**Figure 1:**
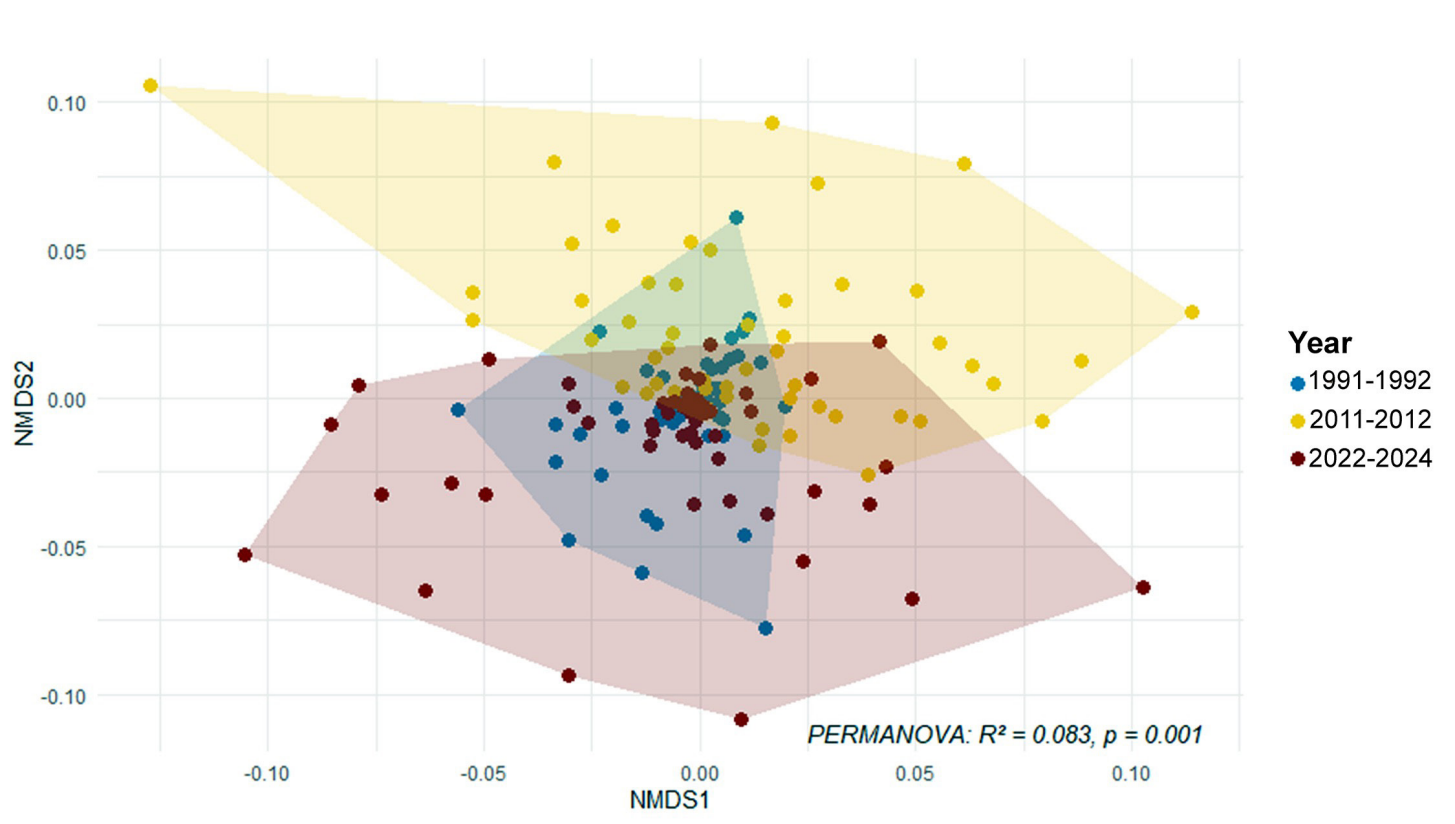
nMDS plot based on Gower distance illustrating the variation in helminth species abundance across different collection periods. Each point represents a sample, and the distance between points reflects dissimilarity in community composition. PERMANOVA results indicate a significant difference in helminth diversity among collection periods (Stress = 0.22).

The nMDS analysis showed greater dissimilarity of helminth abundance for hosts collected between 2011–2012 and 2022–2024 (Figure 1). Furthermore, an overlap of specimens collected in the first collection period (1991–1992) with the other periods was observed, indicating that this period presented similarities in terms of helminth abundance with later collection periods (Figure 1). In fact, although significant, the PERMANOVA result indicates a weak influence of the collection period on the similarity in helminth species abundance among the analyzed hosts, since the low R^2^ value (0.083) suggests that temporal variation accounts for only a minor ecological effect on community structure (p = 0.001).

**Figure 2:**
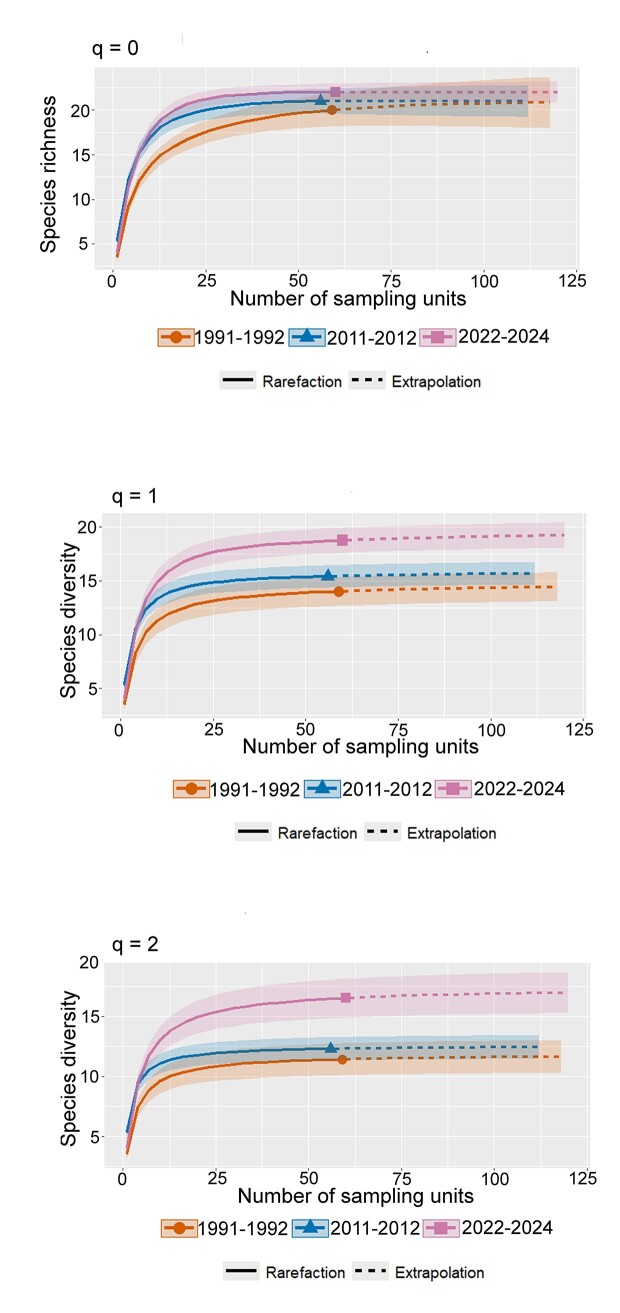
Hill diversity of helminth communities across collection periods. Diversity indices are shown for q = 0 (species richness), q = 1 (Shannon diversity), and q = 2 (Simpson diversity). The 2022–2024 period consistently shows higher diversity compared to 2011–2012 and 1991–1992. Rarefaction and extrapolation curves indicate that these differences persist even when accounting for sampling effort, suggesting that more recent communities are both richer in species and more evenly distributed in abundance.

The analysis of Hill diversity across collection periods revealed a consistent increase in helminth community diversity over time (Figure 2). Species richness (q = 0) was highest in 2022–2024, followed by 2011–2012 and 1991–1992, with rarefaction curves indicating that the more recent period maintained higher diversity even with fewer sampled units (Figure 2). Shannon diversity (q = 1) followed the same pattern, suggesting that the abundance distribution of species is more even in the latest period (Figure 2). Simpson diversity (q = 2) also peaked in 2022–2024, indicating a reduction in dominance by previously common species, although differences among periods were less pronounced for q = 2, reflecting the influence of abundant species (Figure 2). Overall, these results indicate that helminth communities have become more diverse and balanced in species abundance over time.

**Figure 3:**
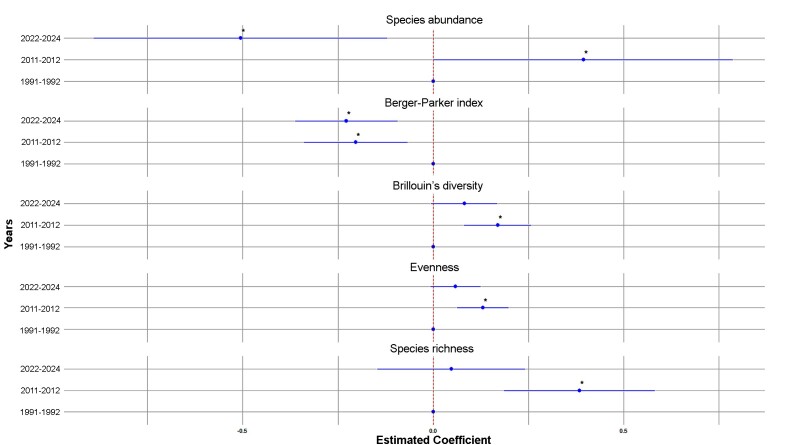
Estimated effects of collection year on helminth community diversity indices. Blue points represent estimated coefficients for each period, with horizontal lines indicating 95% confidence intervals. Asterisks denote statistically significant differences compared to the baseline period (1991–1992). Metrics include species abundance, Berger-Parker Index, Brillouin’s diversity index, evenness, and species richness.

Across all metrics, including species abundance, Berger Parker dominance, Brillouin diversity, evenness, and species richness, temporal changes relative to the 1991–1992 baseline varied among periods (Figure 3). The 2022–2024 period consistently showed higher species abundance and richness, together with lower dominance, suggesting more balanced communities. In contrast, the 2011–2012 period presented greater diversity and evenness. Statistically significant differences are indicated by asterisks (Figure 3).

Tukey’s multiple comparisons revealed significant differences in several helminth diversity metrics across collection periods (Table 4). Total abundance was significantly lower in 2022–2024 compared to 1991–1992 and 2011–2012, with no difference between 1991–1992 and 2011–2012 (Table 4). Species dominance (Berger-Parker index) decreased significantly from 1991–1992 to both 2011–2012 and 2022–2024 but did not differ between 2011–2012 and 2022–2024 (Table 4). Brillouin diversity was higher in 2011–2012 than in 1991–1992, with no other significant differences (Table 4). Evenness increased significantly from 1991–1992 to 2011–2012, while richness increased from 1991–1992 to 2011–2012 and decreased in 2022–2024 relative to 2011–2012, showing no difference between 1991–1992 and 2022_2024 (Table 4). These results indicate temporal changes in helminth community structure, reflecting shifts in species composition and dominance over time.

**Table 4:** Results of pairwise comparisons of parasite communities of *Orthopristis rubra* among samples from the coastal zone of Rio de Janeiro, Brazil, across sampling periods (1991–1992, 2011–2012, and 2022–2024). The table presents metrics for each comparison, including estimated differences (diff) in model coefficients, 95% confidence intervals (CI), and corresponding p-values for total species abundance, Berger–Parker dominance index, Brillouin’s diversity index, evenness, and species richness. Significant results (p < 0.05) indicate temporal changes in parasite community structure.

**Metrics**	**Comparison**	**Difference (diff)**	**95% CI lower**	**95% CI upper**	**p-valor**
**Species abundance**	2010-2011 – 1991-1992	0.3942	-0.0756	0.8641	0.1193
	2022-2024 – 1991-1992	-0.5056	-0.9674	-0.0438	0.0281
	2022-2024 – 2010-2011	-0.8998	-1.3678	-0.4318	0.00003
**Berger-Parker**	2010-2011 – 1991-1992	-0.2035	-0.367	-0.0401	0.0103
	2022-2024 – 1991-1992	-0.2278	-0.3884	-0.0671	0.0028
	2022-2024 – 2010-2011	-0.0242	-0.187	0.1385	0.934
**Brillouin index**	2010-2011 – 1991-1992	0.169	0.0631	0.2748	0.0006
	2022-2024 – 1991-1992	0.0817	-0.0223	0.1857	0.1546
	2022-2024 – 2010-2011	-0.0873	-0.1927	0.0181	0.1259
**Evenness**	2010-2011 – 1991-1992	0.1304	0.0501	0.2107	0.0005
	2022-2024 – 1991-1992	0.0587	-0.0202	0.1377	0.1865
	2022-2024 – 2010-2011	-0.0717	-0.1517	0.0083	0.0888
**Species richnness**	2010-2011 – 1991-1992	0.3835	0.1469	0.6201	0.0005
	2022-2024 – 1991-1992	0.0481	-0.1845	0.2806	0.8768
	2022-2024 – 2010-2011	-0.3354	-0.5711	-0.0998	0.0027

## Discussion

This long-term comparative study revealed clear temporal changes in the structure of the metazoan parasite community of *O. rubra* from Sepetiba Bay, southeastern Brazil. Although the composition of the core parasite fauna remained relatively stable, the relative dominance, diversity indices, and evenness values exhibited significant variation among the three collection periods (1991–1992, 2011–2012, and 2022–2024). These patterns reflect gradual ecological adjustments in host–parasite interactions likely linked to environmental transformations and the progressive recovery of the estuarine ecosystem over the last three decades.

The overall increase in parasite species richness (in 2023–2024) and evenness (in 2011–2012) suggests a partial reorganization of community structure, possibly associated with improved environmental conditions and higher complexity of food webs. Similar patterns have been observed in long-term studies where eutrophication decreased or benthic biodiversity recovered (Virgilio et al., 2023). The persistence of the dominant digenean *I*. *longovatus* across all sampling years indicates a relatively stable trophic pathway involving molluscan and fish intermediate hosts, consistent with previous findings in other Haemulidae from tropical estuaries (Luque & Alves, 2001; Paschoal et al., 2023, 2024). However, its declining relative abundance and the emergence of less common taxa (e.g., *Diphterostomum brusinae*) in recent years may signal changes in transmission efficiency and host diet composition.

Environmental stress, driven by factors such as pollution, habitat alteration, eutrophication, and fluctuations in water quality, is known to reduce parasite diversity and favor generalist or direct-life-cycle taxa (Marcogliese, 2005; Sures et al., 2023). The transient increase in parasite diversity observed here, notably in 2011–2012, might indicate a moderate ecological recovery following earlier degradation events in Sepetiba Bay. Historical records indicate that Sepetiba Bay was subjected to intense industrial discharges and domestic effluents until the early 2000s. From that period onward, the implementation of environmental monitoring programs, increased regulatory enforcement, and mitigation initiatives led to a gradual improvement in water quality and fish community reorganization (Araújo et al., 2002). Parasites with complex life cycles, particularly digeneans, tend to respond positively to ecosystem restoration because their transmission depends on multiple trophic links (Poulin 2004, Palm, 2011; Blasco-Costa & Poulin, 2017). Thus, the observed diversification of the parasite community in *O*. *rubra* is consistent with the recovery of intermediate host populations and the re-establishment of food-web connectivity.

The weak but significant temporal signal detected in the multivariate analyses (nMDS and PERMANOVA) also supports the interpretation that changes in parasite assemblages are subtle and cumulative rather than abrupt. This pattern is typical of estuarine systems undergoing slow environmental improvement (Marcogliese, 2023). Moreover, the significant differences in host size among periods could have influenced infection parameters, since larger or older fish generally harbor more diverse parasite infracommunities due to prolonged exposure and broader diet (Lo et al., 1998; Poulin, 2020). Future analyses integrating fish ontogeny with ecological and environmental indicators could help disentangle the respective roles of environmental versus host-related factors.

From an ecological standpoint, the present findings reinforce the usefulness of fish parasites as bioindicators of ecosystem change. The temporal shift from low-diversity, dominance-heavy communities to more balanced assemblages mirrors trends described for benthic and planktonic communities under moderate recovery conditions (Sures et al., 2023). In this sense, *O. rubra* and its parasite fauna may act as a sentinel system, as changes in parasite community composition and infection patterns can reflect alterations in environmental conditions, thereby providing ecological indicators of long-term health in Sepetiba Bay and other coastal environments exposed to anthropogenic pressures.

Finally, this study highlights the value of maintaining historical parasitological records and continuous monitoring. Long-term data allows distinguishing natural variability from directional trends, improving our understanding of parasite ecology under changing environmental scenarios (Luque & Poulin, 2008; Wood et al., 2023). Integrating such datasets with molecular tools and environmental indicators will be crucial to strengthening the role of parasitology within the broader One Health framework, as host–parasite dynamics can reflect interactions between environmental quality, wildlife health, and potential risks to human populations, thereby supporting more comprehensive aquatic ecosystem management.

## Conclusions

This long-term comparative analysis of metazoan parasite communities in *O. rubra* from Sepetiba Bay demonstrates that parasite assemblages can serve as sensitive indicators of gradual ecological change. Although the core parasite fauna remained relatively stable over more than three decades, shifts in abundance, diversity, and dominance patterns reflect a slow but measurable reorganization of community structure, likely linked to environmental recovery and increased habitat complexity in the bay. The results emphasize the importance of continuous parasitological monitoring to detect subtle ecological responses to anthropogenic and climatic pressures. Integrating these long-term datasets with environmental and molecular indicators will enhance our understanding of ecosystem health and resilience in tropical coastal environments.

## Data Availability

The data are available from the corresponding author on reasonable request.
